# *Ins1* Gene Up-Regulated in a *β*-Cell Line Derived from *Ins2* Knockout Mice

**DOI:** 10.1080/15438600303730

**Published:** 2003

**Authors:** Loïc Leroux, Béatrice Durel, Valérie Autier, Louise Deltour, Danielle Bucchini, Jacques Jami, Rajiv L. Joshi

**Affiliations:** 1 Department of Genetics, Development and Molecular Pathology Institut Cochin, INSERM, CNRS Université René Descartes 24 rue du Faubourg Saint-Jacques Paris 75014 France; 2 MERCK Laboratories Chilly Mazarin France

## Abstract

The authors have derived a new *β*-cell line (βIns2^−/−lacZ^) from Ins2^−/−^ mice that carry the *lacZ* reporter gene under control of the *Ins2* promoter. βIns2^−/−lacZ^ cells stained positively using anti-insulin antibody, expressed *β*-cell–specific genes encoding the transcription factor PDX-1, glucokinase, and Glut-2, retained glucose-responsiveness for insulin secretion, and expressed the *lacZ* gene. Analysis of *Ins1* expression by reverse transcriptase–polymerase chain reaction (RT-PCR) showed that *Ins1* transcripts were significantly raised to compensate for the lack of *Ins2* transcripts in βIns2^−/−lacZ^ cells, as compared to those found in *β*TC1 cells expressing both *Ins1/Ins2*. Thus, transcriptional up-regulation of the remaining functional insulin gene in Ins2^−/−^ mice could potentially contribute to the *β*-cell adaptation exhibited by these mutants, in addition to the increase in *β*-cell mass that we previously reported.We have also shown that *lacZ* expression, as analyzed by determining *β*-galactosidase activity, was up-regulated by incubating βIns2^−/−lacZ^ cells with GLP-1 and/or IBMX, 2 known stimulators of insulin gene expression. These cells thus represent a new tool for testing of molecules capable of stimulating *Ins2* promoter activity


*Experimental Diab. Res.*, 4:7–12, 2003
Copyright © 2003 Taylor & Francis
1543-8600/03 $12.00 + .00
DOI: 10.1080/15438600390214572

### *Ins1* Gene Up-Regulated in a $\beta$-Cell Line Derived from *Ins2* Knockout Mice

**Loïc Leroux,[1] Béatrice Durel,[1] Valérie Autier,[2] Louise Deltour,[1] Danielle Bucchini,[1] Jacques Jami,[1] and Rajiv L. Joshi[1]**

[1]*Department of Genetics, Development and Molecular Pathology, Institut Cochin, INSERM, CNRS, Université René Descartes, Paris, France*
[2]*MERCK Laboratories, Chilly Mazarin, France*

**The authors have derived a new $\beta$-cell line ($\beta$Ins2$^{-/-lacZ}$) from *Ins2*$^{-/-}$ mice that carry the *lacZ* reporter gene under control of the *Ins2* promoter. $\beta$Ins2$^{-/-lacZ}$ cells stained positively using anti-insulin antibody, expressed $\beta$-cell–specific genes encoding the transcription factor PDX-1, glucokinase, and Glut-2, retained glucose-responsiveness for insulin secretion, and expressed the *lacZ* gene. Analysis of *Ins1* expression by reverse transcriptase–polymerase chain reaction (RT-PCR) showed that *Ins1* transcripts were significantly raised to compensate for the lack of *Ins2* transcripts in $\beta$Ins2$^{-/-lacZ}$ cells, as compared to those found in $\beta$TC1 cells expressing both *Ins1/Ins2*. Thus, transcriptional up-regulation of the remaining functional insulin gene in *Ins2*$^{-/-}$ mice could potentially contribute to the $\beta$-cell adaptation exhibited by these mutants, in addition to the increase in $\beta$-cell mass that we previously reported. We have also shown that *lacZ* expression, as analyzed by determining $\beta$-galactosidase activity, was up-regulated by incubating $\beta$Ins2$^{-/-lacZ}$ cells with GLP-1 and/or IBMX, 2 known stimulators of insulin gene expression. These cells thus represent a new tool for testing of molecules capable of stimulating *Ins2* promoter activity.**

**Keywords** $\beta$-Cell Line; Compensatory Responses; Diabetes; Glucose Homeostasis; Insulin Genes; Knockout Mice

Received 22 August 2002; accepted 20 November 2002.

The authors thank D. Hanahan for providing Rip1-Tag2 mice and F. Radvanyi, M. Kergoat, C. Charron, and M. Asfari for useful discussions. The authors also thank N. Bazin and V. Drouet for their help in transgenic animal facility.

Address correspondence to R. L. Joshi, Département GDPM–Institut Cochin, 24 rue du Faubourg Saint-Jacques, 75014 Paris, France. E-mail: joshi@cochin.inserm.fr

Insulin resistance and $\beta$-cell dysfunction are the two major characteristic features of type 2 diabetes. It is now well admitted that overt diabetes does not develop as long as the $\beta$-cells can secrete increasing amounts of insulin to overcome insulin resistance [1]. Several groups have extensively investigated the molecular mechanisms that regulate insulin gene expression as well as insulin secretion during the past several years [2, 3]. Besides, a few studies in rodents also showed that the $\beta$-cell mass could significantly increase during pregnancy [4] or after glucose infusion for a short period, revealing potential plasticity of the $\beta$-cell compartment [5, 6]. Moreover, the study of knockout mice for insulin receptor substrate (IRS)-1, an intracellular mediator of insulin signaling, provided convincing evidence that insulin resistance could be overcome if $\beta$-cell mass were increased [7]. This conclusion was strongly supported by the observation that insulin resistance in IRS-2–knockout mice, which fail to increase their $\beta$-cell mass, leads to overt diabetes [8].

In recent years, transgenic and gene-targeting approaches in the mouse were extensively applied to examine the effects of manipulating the expression of genes encoding key players in $\beta$-cell development and/or function, such as the transcription factor PDX-1 [9, 10] or proteins involved in glucose-regulated insulin secretion, such as glucokinase (GK) [11] or the glucose transporter Glut-2 [12]. We previously generated knockout mice for the *Ins1* and *Ins2* genes [13]. The single *Ins1*$^{-/-}$ or *Ins2*$^{-/-}$ mutants were viable, fertile, and did not show any major metabolic alteration. Total pancreatic insulin content in *Ins1*$^{-/-}$ or *Ins2*$^{-/-}$ mice was comparable to that found in control animals, indicating that compensatory up-regulations take place in these mutants. Analysis of the transcripts of the remaining functional insulin gene revealed a dramatic increase of *Ins1* transcripts in total pancreatic RNA from *Ins2*$^{-/-}$ mice. Interestingly, morphometric analysis of the pancreases showed that $\beta$-cell mass in both kinds of mutants was significantly augmented, particularly in *Ins2*$^{-/-}$ mutants. Thus, absence of either one of the 2 insulin genes was partly compensated at the tissue level by a specific increase of the $\beta$-cell compartment [13].

It was, however, difficult to determine whether some compensatory up-regulation of *Ins1* could also take place in individual $\beta$-cells in *Ins2*$^{-/-}$ mice. We have addressed this issue here using a $\beta$-cell line derived from *Ins2*$^{-/-}$ mutants. Because *Ins2*$^{-/-}$ mice carry *lacZ* reporter gene under the control of the *Ins2* promoter, such $\beta$-cells also represent an interesting tool for the testing of drugs that could up-regulate *Ins2* promoter activity.

### MATERIALS AND METHODS

### Animals

Generation of *Ins2*$^{-/-}$ mice has been described previously [14]. These mutant mice were crossed with Rip1-Tag2 mice [15] (kindly provided by D. Hanahan) and an *Ins2*$^{-/-}$, Rip1-Tag2 mouse line was established. The offspring were genotyped by polymerase chain reaction (PCR) using specific primers.

### Derivation of the $\beta$Ins2$^{-/-lacZ}$ Cell Line

$\beta$-Cell lines from *Ins2*$^{-/-}$, Rip1-Tag2 mice were isolated as previously described [16]. Briefly, 10-week-old *Ins2*$^{-/-}$, Rip1-Tag2 mice were killed by cervical dislocation, and the pancreases were perfused through the bile duct with 2 mg/mL collagenase (Sigma, St. Louis, MO) dissolved in Hank's buffered saline solution (HBSS; Life Technologies, Gaithersburg, MD). The pancreases were incubated for 20 minutes at 37°C with gentle agitation. The digested material was washed 3 times in HBSS containing 10% fetal bovine serum (FBS; Techgen, Les Ullis, France) and then recovered in RPMI with 10% FBS. Islets were handpicked under a dark-field microscope and cultured in the same medium in an incubator with 5% $CO_2$ at 37°C. Islets were then placed individually in 24-well plates and cultured in high-glucose medium containing 25 mM Hepes (Life Technologies), 15% horse serum (Life Technologies), 2.5% FBS, 200 U/mL penicillin, and 200 $\mu$g/mL streptomycin. After approximately 2 months in culture, islets that clearly expanded were removed, trypsinized, and plated onto 96-well plates to recover $\beta$-cell lines. At confluency, cells were usually split at a 1:2 or 1:3 ratio. $\beta$TC1 cells (obtained from D. Hanahan) used in some experiments were cultured in the same medium.

### Immunocytochemistry and Histochemistry

For insulin detection, cells were fixed in 4% paraformaldehyde, incubated first with guinea pig polyclonal anti-insulin antibody and then with peroxidase-coupled goat anti–guinea pig serum as described [14]. The peroxidase activity was revealed using diaminobenzidine. To detect $\beta$-galactosidase ($\beta$-gal) activity, cells were fixed in 0.25% glutaraldehyde and then stained using 5-bromo-4-chloro-3-indolyl $\beta$-D-galactoside (X-gal) as described [13].

### Reverse Transcriptase (RT)-PCR Analysis of Gene Expression

The primers and probes used to analyze insulin 1/insulin 2, glucagon, somatostatin, pancreatic polypeptic (PP), and $\beta$-actin transcripts have been specified previously [13]. The primer sets and oligonucleotide probes used to analyze the transcripts for PDX-1, GK, Glut-2, and TATA-binding protein (TBP) were as follows: 5′-TCGCTGGGATCACTGGAGCA-3′/5′-GGTCCGCTGTGT AAGCACC-3′ and 5′-[$^{32}$P]-GACC TTTCCCGAATGGAA CC-3′ for PDX-1; 5′-CACCCAACT GCGAAATCACC-3′/5′-CATTTGTGGGGAGTC-5′ and 5′-[$^{32}$P]-GGGCCAGTGAAATCCAGGCA-3′ for GK; 5′-GA GCCAAGGACCCCGTCCTA-3′/5′-GTGAAGACCAGGAC CACCCC-3′ and 5′-[$^{32}$P]-GCCCTCTGCTTCCAGTACAT-3′ for Glut-2; 5′-AAGAGAGCCACGGACAACTG-3′/5′-TACT GAACTGCTGGTGGGTC-3′ and 5′-[$^{32}$P]-GAGTTGTGCA GAAGTTGGGC-3′ for TBP. The following PCR reactions were used: 48°C, 45 minutes; 94°C, 2 minutes, 94°C, 30 seconds; 60°C, 1 minute, 68°C, 2 minutes for 25 cycles; 72°C 10 minutes. The PCR products were run on agarose gels, transferred onto Hybond membranes (Amersham, Saclay, France), and hybridized using [$^{32}$P]-labeled oligonucleotide probes. For insulin 1/insulin 2, a unique primer pair was used for RT-PCR, and the PCR products were digested with MspI before Southern blot analysis. Hybridization using a single [$^{32}$P]-labeled oligonucleotide probe revealed a fragment of 71 bp for insulin 1 and another of 112 bp for insulin 2. Quantification of PCR products was performed using a Phosphorimager equipped with ImageQuant software (Molecular Dynamics) and was expressed in relation to the internal control.

### Total Cellular Insulin Content and Insulin Secretion

Total cellular insulin content was obtained by radioimmunoassay (RIA) kit (ICN, Irvine, CA) using acid/alcohol extracts as described [16]. Insulin secretion assays were performed with cells cultured in 48-well plates when they reached 70% to 80% confluency as described [15]. Briefly, cells were preincubated for 1 hour in Krebs-Ringer buffer, then incubated for 2 hours in Krebs-Ringer buffer containing different glucose concentrations as indicated. The secretion medium was recovered, centrifuged for 10 minutes at 1000 rpm, and supernatants were stored at $-80^\circ$C until used for determining insulin levels by RIA.

### Determination of $\beta$-Gal Activity

Cells were cultured in 48-well plates in high-glucose medium and incubated for 48 hours in the presence of GLP-1 (Sigma) and/or 3-isobutyl-1-methylxanthine (IBMX) (Sigma) at the indicated concentrations. Cells were then washed with phosphate-buffered saline (PBS) solution and lysed in a commercial lysis buffer (Boehringer, Mannheim, Germany). $\beta$-Gal activity in the protein extracts was determined by colorimetric assay using o-nitrophenyl $\beta$-D-galactopyranoxide (ONPG) (Sigma) as substrate. Blank values obtained with lysates from $\beta$TC1 cells were substracted.

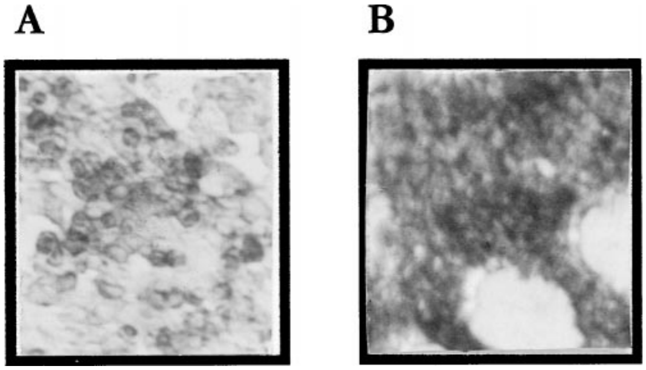

**FIGURE 1**
Immunocytochemical detection of insulin (*A*) and histochemical analysis of *lacZ* expression (*B*) in $\beta$Ins2$^{-/-\text{lacZ}}$ cells. (*A*) Cells were fixed and stained using an anti-insulin antibody. (*B*) Cells were fixed and $\beta$-gal activity was visualized by X-gal staining. Magnification: 10×.

### RESULTS AND DISCUSSION

### $\beta$-Cell Line Derived From *Ins2*$^{-/-}$ Mice

In previous studies, $\beta$-cell lines were isolated from islets of transgenic mice expressing SV40 large T antigen gene under the control of rat *Ins1* gene promoter (Rip1-Tag2 mice) [15, 16]. Therefore *Ins2*$^{-/-}$ mice were crossed with Rip1-Tag2 mice and an *Ins2*$^{-/-}$, Rip1-Tag2 mouse line was established. Hyperplastic islets from pancreases of *Ins2*$^{-/-}$, Rip1-Tag2 mice were isolated prior to tumor formation, i.e., at 8 to 10 weeks. After a 2-month culture in high-glucose medium (22 mM), some islets clearly expanded as a result of efficient proliferation of $\beta$-cells transformed by SV40 T antigen, and individually trypsinized. One of the $\beta$-cell lines recovered from cultured cells used in this study was designated $\beta$Ins2$^{-/-\text{lacZ}}$. These cells showed a doubling time of 10 days and grew in islets-like clusters. The cells were not further subcloned to avoid clonal effects and used up to passage 19 in the experiments described here.

We first confirmed that $\beta$Ins2$^{-/-\text{lacZ}}$ cells were $\beta$-cells. $\beta$Ins2$^{-/-\text{lacZ}}$ cells were stained using an anti-insulin antibody (Figure 1*A*). *Ins2*$^{-/-}$ mice carry the *lacZ* reporter gene under control of the *Ins2* promoter and the presence of $\beta$-gal in $\beta$Ins2$^{-/-\text{lacZ}}$ cells was detected by X-gal staining (Figure 1*B*). Because the explanted islets of origin also contained $\alpha$, $\delta$, and PP cells, which produce glucagon, somatostatin, and pancreatic polypeptide, respectively, we checked for the expression of the corresponding genes for these hormones by RT-PCR, using total RNA from $\beta$Ins2$^{-/-\text{lacZ}}$ cells. No transcript was found for somatostatin and pancreatic polypeptide. Few glucagon transcripts were detected (not shown), as is frequently observed when deriving a $\beta$-cell line using Rip1-Tag2 construct [17].

We also analyzed the expression of genes encoding PDX-1, GK, and Glut-2. PDX-1 is a transcription factor that is essential for pancreas development and in the regulation of islet gene expression in mature $\beta$-cells [18]. It was shown that PDX-1 expression in the liver upon adenoviral vector–mediated gene transfer in the mouse resulted in transdifferentiation of a small fraction of hepatocytes into insulin-secreting cells [19]. GK is the high-$K_M$ hexokinase present in both $\beta$-cells as well as in hepatocytes and catalyzes the initial conversion of glucose to glucose-6-phosphate. Finally, Glut-2 is a glucose transporter also present in $\beta$-cells and hepatocytes [20]. The role of GK and Glut-2 in glucose sensing was fully confirmed by global and tissue-specific disruption of the corresponding genes in the mouse. Knockout mice for GK or Glut-2 presented defective insulin secretion and developed diabetes [11, 12]. As presented in Figure 2, transcripts for PDX-1, GK, and Glut-2 could be readily detected by RT-PCR using total RNA from $\beta$Ins2$^{-/-\text{lacZ}}$ cells.

In conclusion, $\beta$Ins2$^{-/-\text{lacZ}}$ cells continue to synthesize insulin and express important $\beta$-cell–specific marker genes. Moreover, *Ins2* promoter drives expression of *lacZ* gene in these cells.

### Glucose-Induced Insulin Secretion From $\beta$Ins2$^{-/-\text{lacZ}}$ Cells

We examined the ability of $\beta$Ins2$^{-/-\text{lacZ}}$ cells to secrete insulin in response to glucose because this property is frequently lost when deriving a $\beta$-cell line [17]. We first measured total cellular insulin content in acid-alcohol extracts

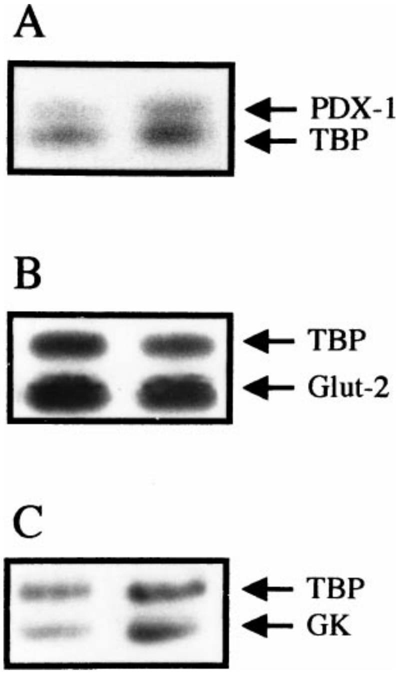

**FIGURE 2**
RT-PCR analysis of $\beta$-cell–specific gene expression. Transcripts for PDX-1 (*A*), Glut-2 (*B*), and GK (*C*) were amplified using total RNA from $\beta$Ins2$^{-/-\mathrm{lacZ}}$ cells and analyzed by Southern blotting. TBP mRNA was coamplified as control. The results of 2 separate samples are presented. The sizes of various PCR products correspond to 275 bp (PDX-1), 150 bp (Glut-2), 162 bp (GK), and 233 bp (TBP).

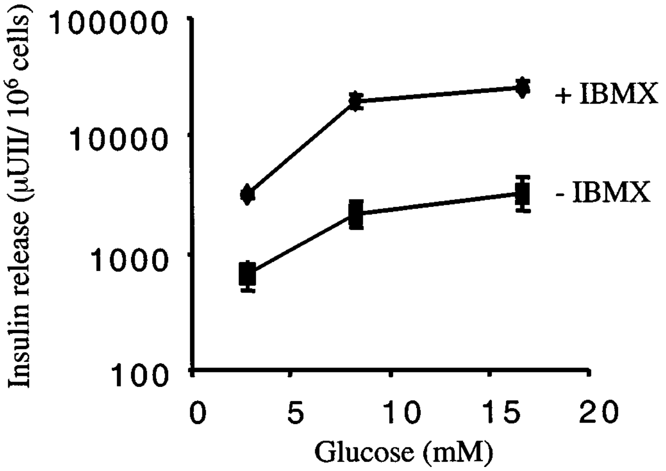

**FIGURE 3**
Glucose-induced insulin secretion in $\beta$Ins2$^{-/-\mathrm{lacZ}}$ cells. Insulin release was measured in the absence or in the presence of 0.5 mM IBMX. The incubation time was 2 hours. Values are means ± SE from triplicate assays.

of $\beta$Ins2$^{-/-\mathrm{lacZ}}$ cells by RIA. The values obtained (30.04 ± 2.167 mUI/10$^6$ cells) were comparable to those previously reported with other murine $\beta$-cell lines in which both the *Ins1* and *Ins2* genes were functional [15, 16]. Thus, increased *Ins1* gene expression appears to quantitatively compensate the absence of insulin transcripts from *Ins2*-null alleles in $\beta$Ins2$^{-/-\mathrm{lacZ}}$ cells. Glucose-induced insulin secretion from $\beta$Ins2$^{-/-\mathrm{lacZ}}$ cells was subsequently analyzed by incubating the cells in Krebs-Ringer secretion buffer supplemented with 2.8, 8.3, or 16.7 mM glucose. After incubation for 2 hours, the amount of insulin released in the medium was determined by RIA and the results obtained are presented in Figure 3. Insulin release from $\beta$Ins2$^{-/-\mathrm{lacZ}}$ cells increased with increasing glucose concentrations. The values obtained at 16.7 mM glucose were about 5-fold higher as compared to those at 2.8 mM glucose. Insulin secretion from $\beta$Ins2$^{-/-\mathrm{lacZ}}$ cells was also examined in the presence of IBMX, which is known to potentiate glucose-stimulated insulin release from $\beta$-cells. Addition of 0.5 mM IBMX in the secretion buffer enhanced insulin release from $\beta$Ins2$^{-/-\mathrm{lacZ}}$ cells at all glucose concentrations (Figure 3). In the presence of IBMX, insulin secretion at high glucose concentration (16.7 mM) was ~8.3-fold higher than that at low glucose concentration (2.8 mM). All these results indicate that $\beta$Ins2$^{-/-\mathrm{lacZ}}$ cells have retained glucose responsiveness for insulin secretion.

### *Ins1* Gene is Up-Regulated in $\beta$Ins2$^{-/-\mathrm{lacZ}}$ Cells

To investigate possible up-regulation of the *Ins1* gene in the absence of *Ins2* transcripts in $\beta$Ins2$^{-/-\mathrm{lacZ}}$ cells, we compared insulin transcripts produced in $\beta$Ins2$^{-/-\mathrm{lacZ}}$ cells with those in $\beta$TC1 cells. This latter cell line was derived from Rip1-Tag2 mice in which both the *Ins1* and *Ins2* genes are functional. Insulin gene expression was analyzed by RT-PCR using total cellular RNA. In $\beta$TC1 cells, *Ins1* transcripts represent the minority of insulin transcripts (Figure 4*B*). In $\beta$Ins2$^{-/-\mathrm{lacZ}}$ cells, the amounts of *Ins1* transcripts were significantly higher than those found in $\beta$TC1 cells (Figure 4*A*). Quantitative analysis of RT-PCR products for insulin as well as $\beta$-actin transcripts showed that *Ins1* transcripts in $\beta$Ins2$^{-/-\mathrm{lacZ}}$ cells represent 55% to 88% of total insulin transcripts present in $\beta$TC1 cells. These results indicate that the *Ins1* gene is up-regulated in $\beta$Ins2$^{-/-\mathrm{lacZ}}$ cells to compensate for the absence of *Ins2* transcripts. It can be inferred from this study that *Ins1* gene up-regulation could also potentially take place in individual $\beta$-cells in vivo in *Ins2*$^{-/-}$ mice, in the compensating responses exhibited by these mutants to maintain normal glucose homeostasis [14].

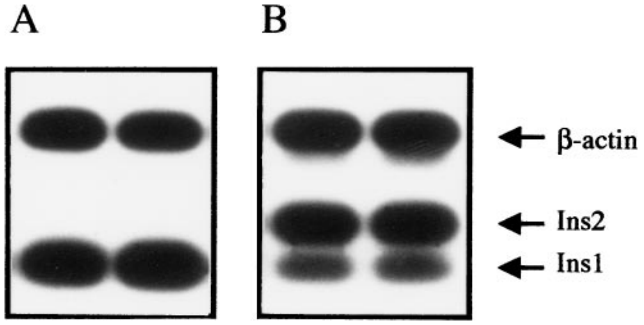

**FIGURE 4**
RT-PCR analysis of insulin gene expression in $\beta$Ins2$^{-/-\text{lacZ}}$ (*A*) and $\beta$TC1 (*B*) cells. Transcripts for Insulin 1 (Ins1)/Insulin 2 (Ins2) were amplified using total RNA from $\beta$Ins2$^{-/-\text{lacZ}}$ or $\beta$TC1 cells and analyzed by Southern blotting. $\beta$-Actin mRNA was coamplified as control. The results of 2 separate samples are presented. The sizes of various PCR products correspond to 76 bp (Insulin 1), 112 bp (Insulin 2), and 243 bp ($\beta$-actin).

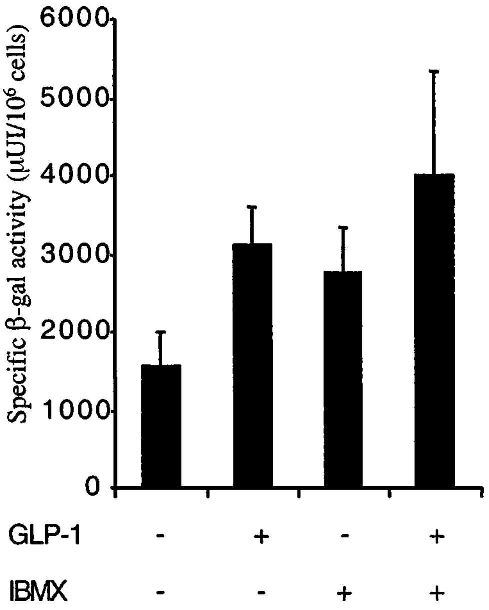

**FIGURE 5**
Analysis of $\beta$-gal activity in $\beta$Ins2$^{-/-\text{lacZ}}$ cells cultured under various conditions. $\beta$-Gal activity was determined in $\beta$Ins2$^{-/-\text{lacZ}}$ cells cultured in high-glucose medium (22 mM) supplemented or not for 48 hours with $10^{-7}$ M GLP-1 and/or 0.5 mM IBMX. A blank value obtained with $\beta$TC1 cells was substracted. Values are means $\pm$ SE from triplicate and quadruplicate assays.

### Induction of the *Ins2-lacZ* Gene in $\beta$Ins2$^{-/-\text{lacZ}}$ Cells

Regulation of insulin gene expression has been examined in many studies using different $\beta$-cell lines transfected with reporter genes under control of rodent or human insulin promoter. In some of these studies, several-fold induction of insulin promoter activity by glucose was reported [21, 22]. Because $\beta$Ins2$^{-/-\text{lacZ}}$ cells express the *lacZ* gene inserted at the *Ins2* locus, these cells represent an interesting tool for testing or screening of molecules that can up-regulate *Ins2* promoter activity, because *lacZ* expression can be easily monitored by measuring $\beta$-gal activity in cellular extracts. We could not examine the effect of glucose on *Ins2-lacZ* expression, because only a moderate decrease, if any, of $\beta$-gal activity was observed when $\beta$Ins2$^{-/-\text{lacZ}}$ cells grown in high glucose were transferred to low-glucose medium (not shown). We could, however, test the effect of GLP-1 and IBMX on *Ins2-lacZ* gene expression in $\beta$Ins2$^{-/-\text{lacZ}}$ cells. GLP-1 is a hormone secreted by the gut during digestion and has been shown to stimulate insulin gene transcription under hyperglycemic conditions [23]. IBMX has also been reported to stimulate insulin gene expression [24].

As shown in Figure 5, incubation of $\beta$Ins2$^{-/-\text{lacZ}}$ cells cultured in high-glucose medium with $10^{-7}$ M GLP-1 for 2 days resulted in 97% increase in $\beta$-gal activity as compared to $\beta$Ins2$^{-/-\text{lacZ}}$ cells cultured without GLP-1. A similar increase in $\beta$-gal activity was also observed for $\beta$Ins2$^{-/-\text{lacZ}}$ cells incubated with 0.5 mM IBMX. The $\beta$-gal activity in $\beta$Ins2$^{-/-\text{lacZ}}$ cells cultured in the presence of both GLP-1 and IBMX was increased up to 157% as compared to untreated cells. These results demonstrate that the *Ins2* promoter can be induced in $\beta$Ins2$^{-/-\text{lacZ}}$ cells.

In conclusion, the advantage of such a model $\beta$Ins2$^{-/-\text{lacZ}}$ cell line is twofold: (1) to understand the transcriptional network governing the expression of the *Ins2* promoter, and (2) to study the mechanisms involved in the up-regulation of *Ins1* promoter activity. These cells also represent an interesting new tool for the screening of molecules that could stimulate *Ins2* promoter activity under high-glucose conditions, and therefore would have potential therapeutic interest for type 2 diabetes, because the *Ins2* gene in the mouse is homologous to the human insulin gene.

### REFERENCES

[1] Kahn, B. B. (1998) Type 2 diabetes: When insulin secretion fails to compensate for insulin resistance. *Cell,* **92,** 593–596.
[2] Docherty, K., and Clark, A. R. (1994) Nutrient regulation of insulin gene expression. *FASEB J.,* **8,** 20–27.
[3] Dumonteil, E., and Philippe, J. (1996) Insulin gene: Organisation, expression and regulation. *Diabetes Metab.,* **22,** 164–173.
[4] Nieuwenhuizen, A. G., Schuiling, G. A., Moes, H., and Koiter, T. R. (1997) Role of increased insulin demand in the adaptation of the endocrine pancreas to pregnancy. *Acta Physiol. Scand.,* **159,** 303–312.

[5] Bernard, C., Thibault, C., Berthault, M. F., Magnan, C., Saulnier, C., Portha, B., Pralong, W. F., Penicaud, L., and Ktorza, A. (1998) Pancreatic beta-cell regeneration after 48-h glucose infusion in mildly diabetic rats is not correlated with functional improvement. *Diabetes,* **47,** 1058–1065.

[6] Bonner, W. S., Deery, D., Leahy, J. L., and Weir, G. C. (1989) Compensatory growth of pancreatic beta-cells in adult rats after short-term glucose infusion. *Diabetes,* **38,** 49–53.

[7] Araki, E., Lipes, M. A., Patti, M. E., Bruning, J. C., Haag, B. R., Johnson, R. S., and Kahn, C. R. (1994) Alternative pathway of insulin signalling in mice with targeted disruption of the IRS-1 gene. *Nature,* **372,** 186–190.

[8] Withers, D. J., Gutierrez, J. S., Towery, H., Burks, D. J., Ren, J. M., Previs, S., Zhang, Y., Bernal, D., Pons, S., Shulman, G. I., Bonner, Weir, S., and White, M. F. (1998) Disruption of IRS-2 causes type 2 diabetes in mice. *Nature,* **391,** 900–904.

[9] Jonsson, J., Carlsson, L., Edlund, T., and Edlund, H. (1994) Insulin-promoter-factor 1 is required for pancreas development in mice. *Nature,* **371,** 606–609.

[10] Marshak, S., Ben-Shushan, E., Shoshkes, M., Havin, L., Cerasi, E., and Melloul, D. (2001) Regulatory elements involved in human pdx-1 gene expression. *Diabetes,* **50(Suppl 1),** S37–S38.

[11] Bali, D., Svetlanov, A., Lee, H. W., Fusco-DeMane, D., Leiser, M., Li, B., Barzilai, N., Surana, M., Hou, H., and Fleischer, N. (1995) Animal model for maturity-onset diabetes of the young generated by disruption of the mouse glucokinase gene. *J. Biol. Chem.,* **270,** 21464–21467.

[12] Guillam, M. T., Hummler, E., Schaerer, E., Yeh, J. I., Birnbaum, M. J., Beermann, F., Schmidt, A., Deriaz, N., Thorens, B., and Wu, J. Y. (1997) Early diabetes and abnormal postnatal pancreatic islet development in mice lacking Glut-2. *Nat. Genet.,* **17,** 327–330.

[13] Leroux, L., Desbois, P., Lamotte, L., Duvillié, B., Cordonnier, N., Jackerott, M., Jami, J., Bucchini, D., and Joshi, R. L. (2001) Compensatory responses in mice carrying a null mutation for Ins1 or Ins2. *Diabetes,* **50(Suppl 1),** S150–S153.

[14] Duvillié, B., Cordonnier, N., Deltour, L., Dandoy, Dron F., Itier, J. M., Monthioux, E., Jami, J., Joshi, R. L., and Bucchini, D. (1997) Phenotypic alterations in insulin-deficient mutant mice. *Proc. Natl. Acad. Sci. U.S.A.,* **94,** 5137–5140.

[15] Efrat, S., Leiser, M., Surana, M., Tal, M., Fusco-Demane, D., and Fleischer, N. (1993) Murine insulinoma cell line with normal glucose-regulated insulin secretion. *Diabetes,* **42,** 901–907.

[16] Radvanyi, F., Christgau, S., Baekkeskov, S., Jolicoeur, C. and Hanahan, D. (1993) Pancreatic beta cells cultured from individual preneoplastic foci in a multistage tumorigenesis pathway: A potentially general technique for isolating physiologically representative cell lines. *Mol. Cell. Biol.,* **13,** 4223–4232.

[17] Levine, F. (1997) Gene therapy for diabetes: Strategies for beta-cell modification and replacement. *Diabetes Metab. Rev.,* **13,** 209–246.

[18] Yamaoka, T., and Itakura, M. (1999) Development of pancreatic islets. *Int. J. Mol. Med.,* **3,** 247–261.

[19] Ferber, S., Halkin, A., Cohen, H., Ber, I., Einav, Y., Goldberg, I., Barshack, I., Seijffers, R., Kopolovic, J., Kaiser, N., and Karasik, A. (2000) Pancreatic and duodenal homeobox gene 1 induces expression of insulin genes in liver and ameliorates streptozotocin-induced hyperglycemia. *Nat. Med.,* **6,** 568–572.

[20] Rolland, F., Winderickx, J., and Thevelein, J. M. (2001) Glucose-sensing mechanisms in eukaryotic cells. *Trends Biochem. Sci.,* **26,** 310–317.

[21] de Vargas, L., Sobolewski, J., Siegel, R., and Moss, L. G. (1997) Individual beta cells within the intact islet differentially respond to glucose. *J. Biol. Chem.,* **272,** 26573–26577.

[22] Leibiger, B., Moede, T., Schwarz, T., Brown, G. R., Kohler, M., Leibiger, I. B., and Berggren, P. O. (1998) Short-term regulation of insulin gene transcription by glucose. *Proc. Natl. Acad. Sci. U.S.A.,* **95,** 9307–9312.

[23] Drucker, D. J. (1998) Glucagon-like peptides. *Diabetes,* **47,** 159–169.

[24] Kohnert, K. D., Ziegler, B., Hahn von Dorsche, H., Hehmke, B., and Schroder, D. (1982) Effects of 3-isobutyl-1-methylxanthine on neonatal pancreatic islets maintained in tissue culture. *Mol. Cell. Endocrinol.,* **28,** 425–437.

